# Stakeholders’ Perspectives on the Current Status of Partnerships between the Food Banking and Healthcare Systems to Address Food Insecurity in the U.S.

**DOI:** 10.3390/nu13124502

**Published:** 2021-12-16

**Authors:** Eminet Abebe Gurganus, Nana Yaa A. Marfo, Marlene B. Schwartz, Kristen Cooksey Stowers

**Affiliations:** 1 Rudd Center for Food Policy & Obesity, University of Connecticut, Hartford, CT 06103, USA; eminet.gurganus@uconn.edu (E.A.G.); marlene.schwartz@uconn.edu (M.B.S.); 2 Department of Human Development and Family Sciences, University of Connecticut, Storrs, CT 06269, USA; 3 Department of Psychological Sciences, University of Connecticut, Storrs, CT 06269, USA; nana.marfo@uconn.edu; 4 Department of Allied Health Sciences, University of Connecticut, Storrs, CT 06269, USA

**Keywords:** social determinants of health (SDOH), food banking, healthcare, food insecurity

## Abstract

One in eight people in the U.S. experience food insecurity (FI). To date, the food banking sector has been at the forefront of efforts to address FI, but the healthcare sector is becoming increasingly involved in such efforts. The extent of collaboration between the two sectors remains unclear. We explored food banking stakeholders’ views on the current state of partnerships between the two sectors. We used purposive sampling to recruit ten key informants for semi-structured interviews. We also conducted a national online survey to gather data from food bank directors (*n* = 137). Thematic analysis generated two major themes: (1) Healthcare and food banking stakeholders are coordinating to achieve collective impact, and (2) Food banking-healthcare partnerships are leveraging various resources and vested interests within the medical community. We found evidence of ongoing partnerships between the two sectors and opportunities to strengthen these partnerships through the support of backbone organizations.

## 1. Introduction

Food insecurity, defined as having limited access to adequate food due to a lack of money and resources, is a major public health concern affecting 38.3 million people in the U.S. [1]. A growing body of literature has found associations between food insecurity and a wide range of negative health outcomes, including hypertension and diabetes [2,3]. Food insecurity has also been linked to poor management of diabetes [4] and higher healthcare utilization and cost [5]. In addition, food insecurity has been associated with obesity in women [6] and with psychosocial stressors and poorer mental health [7].

Although federal food programs provide critical support for food-insecure families, 46 million people each year turn to the food banking sector, a nationwide network of 200 food banks and 49,000 food pantries that provide free groceries for families in need [8,9]. Traditionally, client health outcomes have been overlooked, with food banking organizations focused on their historical purpose-food distribution [10]. In light of the link between food insecurity and health outcomes, however, it has become clear that the food banking system can play a broader role in promoting clients’ health [9,11]. In addition, the food banking system is starting to address the social determinants of health (SDOH) and root causes of food insecurity, such as unemployment, education, transportation, housing, and the built environment [12].

Simultaneously, the healthcare system, which includes stakeholders such as clinical care providers, hospitals, and insurers, is increasingly turning its attention to the need to mitigate adverse social determinants of health (SDOH), including food insecurity [13]. For example, Stenmark and colleagues [13] describe an intervention at two Kaiser Permanente pediatric clinics that screened families for food insecurity and referred those with a positive screen to federal assistance programs and/or community-based nutrition programs. Similarly, some healthcare organizations offer nutritional counseling, connection to onsite or partner food pantries, and fresh fruit and vegetable distribution [14]. This has been fueled by the recognition that only about 20% of health outcomes can be attributed to clinical care and that the remaining 80% depend on health behaviors and upstream social or economic factors [15]. Moreover, healthcare practitioners now recognize that addressing these upstream factors requires multi-sector collaboration [16]. The collective impact framework—defined as “the commitment of a group of important actors from different sectors to a common agenda for solving a specific social problem”—has gained traction as a multi-sector approach to address SDOH [17].

Given the food banking and healthcare systems’ interest and commitment to mitigating food insecurity and associated social determinants, enhanced collaboration between the two systems has the potential to yield greater efficiencies and more positive outcomes. Although both systems are implementing initiatives to combat food insecurity, little is known about how they may be partnering in this endeavor. In the present study, we examined food banking stakeholders’ views on the current state of partnerships between food banking and healthcare systems to address the social determinants of food insecurity. 

## 2. Methods

The study utilized both semi-structured interviews and open-ended responses from a national survey of food bank directors. The University of Connecticut Institutional Review Board determined that this study (IRB approval number: X18-025) does not meet the definition of “human subjects research”.

### 2.1. Semi-Structured Interviews

We conducted in-depth, semi-structured interviews with stakeholders who influence food bank operations. Interviews lasted from 45 min to 1 h. Participants were recruited using a purposive sampling approach to identify “information-rich” stakeholders that are knowledgeable about the topic [18]. Individuals were invited to participate if they were familiar with the food banking system in their professional capacity and matched a list of priority stakeholders and perspectives for the present study (e.g., food bank director, elected official, advocate, funder, researcher). We utilized a referral process to solicit interviewees’ input regarding additional potential participants. A purposive sampling strategy was well-suited to our study, as it allowed us to leverage the diverse geographic locations and professional perspectives among food baking stakeholders. We recruited participants until we reached saturation; determined by the recurrence of themes and no new information emerging from additional interviews [19]. All participants provided verbal consent to be interviewed and audio-recorded before the interview.

A semi-structured interview guide was developed by KCS to outline the topics to be covered in the interview. The guide was adapted from the Center for Social Inclusion’s guidance on “Asking Structural Questions to Identify Structural Entry Points” [20]. The interview covered a broad set of topics related to the food banking system and health inequities among individuals experiencing food insecurity, including structural factors (e.g., policies, funding, access to healthy foods, etc.) and social factors (e.g., discrimination, language barriers, identification requirements, etc.) in the food banking system that potentially contribute to health disparities. As part of the interview, participants were prompted to respond to two questions that are relevant to the present study: (1) Name at least three (and no more than six) institutions impacting structural inequities in the food banking system that contribute to avoidable health disparities and (2) Which institutions impact the root causes of the problem (i.e., health inequities among clients)?

Data were transcribed verbatim and coded in Nvivo version 11 (QSR International, Doncaster, Australia). Authors KCS and NYM read and reread the transcripts for familiarization and developed an initial coding guide through an iterative process. After the first coding round was completed, minor revisions were made to the coding guide. Two coders (NYM and EAG) coded all 10 interviews and regularly consulted with a third coder (KCS) for quality control and reconciliation. Coders reached adequate inter-rater reliability for the first three interviews (Cohen’s Kappa = .81) before coding the remaining interviews [21]. The themes were then analyzed using a general inductive approach [22]. For this analysis of qualitative data from food bank system stakeholders, we focused on the codes that identified the healthcare system as an institutional stakeholder in addressing diet-related health inequities.

### 2.2. National Food Bank Director Survey

Following the completion of the semi-structured interviews, we conducted a national online survey to gather data from a larger group of food banking stakeholders. Specifically, we developed survey questions to collect additional information on the themes identified in the semi-structured interviews. Qualtrics survey links were sent to 310 food bank directors (including all 200 food banks and 77 “food distribution organizations” that are part of the Feeding America national network, as well as 33 of 51 independent food banks for which we could find contact information). Feeding America collaborated with the research team on the development of the semi-structured interview protocol and survey questions, review and interpretation of semi-structured interview data, and promotion of the survey to food bank directors in the network. Each participant provided informed consent before participating. As an incentive, each survey respondent was entered into a raffle to win one of three $500 cash prizes to donate to a food bank of their choice. The survey included 22 open-ended questions. For the purposes of this study, we examined participants’ responses to the following questions: “Does your food bank partner with the medical community for food security screenings or health screenings? If yes, please describe.” Answers to open-ended questions were compiled and analyzed using a content analysis approach [23]. The content analysis allowed the research team to analyze the frequency of specific responses (e.g., number of respondents who mentioned a healthcare-food banking collaboration) in the open-ended text. To test for statistically significant differences between the total sample of survey participants vs. the subsample of participants that responded to the open-ended question describing their food bank’s partnerships with the medical community, we used the prtesti command in Stata 16 software (StataCorp, College Station, TX, USA). The analysis was intended to assess the representativeness of the subsample when compared to the full sample. 

## 3. Results

We interviewed 10 key informants, including a food bank board member, two food bank executive directors, a national anti-hunger organization leader, two foundation program officers, an academic researcher, two anti-hunger advocates, and a state house representative. Participants represented diverse geographic regions.

The response rate for the national food bank director survey was 44%. When asked whether their food bank collaborates with the medical community to conduct food insecurity and health screening, 72 (53%) of survey participants answered “yes”. Of those who answered “yes”, 58 food bank directors then responded to the subsequent open-ended question asking them to describe their partnerships with the medical community. There were no statistically significant differences between this subsample and the full sample of survey respondents in terms of length of employment at the food bank, state, and U.S. region.

Two major themes emerged from the data: (1) Stakeholders from the healthcare and food banking systems are currently coordinating to achieve collective impact on addressing SDOH, and (2) Food bank-healthcare partnerships are leveraging a variety of resources and vested interests within the medical community (Please refer to Table 1). Below, we describe each sub-theme.

### 3.1. Stakeholders from the Healthcare and Food Banking Systems Are Currently Coordinating to Achieve Collective Impact on Addressing SDOH

#### 3.1.1. Collaborating to Address SDOH

Supporting evidence from interviews

Interviewees argued that the same social and economic conditions that underlie food insecurity also underlie healthcare-related challenges, noting, “Part of the reason people don’t have food is that they don’t have a living wage job, they don’t have affordable health and childcare.” In describing how healthcare and food banking partnerships address social determinants, a participant stated:

As people start to look at how we even tackle these root causes, …having connections to people who do SNAP (Supplemental Nutrition Assistance Program), health care screenings, or signing up for health insurance within the food pantries…, those types of initiatives are trying to get at some of these root causes. As opposed to just focusing on pounds of food.

Along the same lines, another participant noted how food pantries are serving as a hub of services that engages healthcare partners, as she said:

[The food pantry]’s like a hub. Hunger is in the middle, just like the middle of a wheel, and from that, I have health, that’s a result of hunger. A lot of the issues within poverty will relate to food insecurity. So...do we fix that problem of poverty by just giving them food? No. We cannot and have not. …why not have somebody at the food bank that hooks them up with adult education in the area. …Have you signed up for all of the things you are due?

Supporting evidence from surveys

Content analysis of open-ended survey responses provided additional evidence to support the existence of collaborations between the food banking and healthcare sectors. The survey revealed extensive associations centered on food insecurity, health screenings, and referrals. Seventeen respondents indicated that they collaborate with community hospitals and/or clinics. One participant mentioned, “We have worked with the local medical community to add questions about food access at intake and provide information on where to get food assistance.” Nine directors reported that their food banks conduct brief health screenings. One director described such an initiative by stating, “We have screened for diabetes with a finger stick and referred to our diabetes pantry. Physicians and nurses have also referred to our diabetes pantry.” Seven directors reported conducting screenings through mobile food pantry programs. 

In explaining the variety of health screening activities that take place at mobile food pantries, one participant mentioned, “We host a mammography mobile [screening] twice a year,” while 14 others mentioned, “diabetes screening” (*n* = 5) and general “health screenings” (*n* = 9). Twenty food bank directors also highlighted screening initiatives targeting specific vulnerable populations. For example, one participant reported that they “partner with health departments to provide Seniors in CSFP (Commodity Supplemental Food Program) with health screenings during food pantry distribution.” Moreover, seven directors explained that they work with healthcare organizations to conduct food insecurity screenings. A participant stated that their food bank will “implement food insecurity screening at three local health systems in order to distribute diabetes-friendly food boxes.” Another participant mentioned that “the local hospital and the local health department come to [food] distributions to offer health screenings.” Seven participants also described the processes in place to facilitate food bank-healthcare partnerships in conducting food insecurity screenings. For instance, one noted:

We are working with two healthcare groups that are implementing food insecurity screenings. We send SNAP screeners to some of those clinics. We are working on establishing a referral system to pantries and a referral program whereby providers can send patient referrals to our Benefits Outreach department through their EMR (Electronic Medical Record). Our Benefits Outreach staff then follows up with those patients to connect them to SNAP and local pantries.

#### 3.1.2. Applying a Collective Impact Framework

Supporting evidence from interviews

Participants described several initiatives at the interface of the healthcare and food banking systems that are utilizing a collective impact approach to mitigate the impact of negative SDOH. Specifically, interviewees detailed instances in which healthcare and food banking partners are working towards a common agenda and shared measurement of success facilitated by a backbone support organization—three central elements of the collective impact framework—to address the root causes of hunger. For example, one participant mentioned:

Another strategy that addresses health equity is our collaborating for clients’ collective impact work. We’ve taken five or six food bank communities and have helped lead them [in] doing a community needs assessment with their partners, helping them to identify what, collectively, they want to make improvements on, what are their measurements, and what role the food bank is going to play now that they have identified those indicators.

Participants identified additional sectors that partner with the food banking and healthcare systems to address SDOH. One participant referenced a grant-funded partnership between a food bank, local healthcare providers, plus housing and community development organizations, in which the food bank is serving as the lead organization:

The REDACTED Food Bank (partnering with Feeding America), got grant funding to look at how we build collective impact. Starting with the food bank being the backbone organization, but partnering with other groups in the community who are also dealing with those root cause issues and getting, maybe it’s Urban League involved, or maybe it’s Habitat for Humanity or other groups, again hospitals, health insurance companies, to partner together so that you don’t have silos…

Food policy councils were mentioned as a key avenue via which collective impact collaborations between the healthcare and food banking sectors occur. A participant described food policy councils as “[organizations] bringing in the private sector, the public sector, the public health sector, …and community members to shape decisions being made and recommendations for action and innovations.

Supporting evidence from surveys

Survey respondents similarly highlighted collective impact initiatives focused on training and capacity building to facilitate healthcare and food banking relationships. One respondent mentioned, “We have our first MD (medical doctor) on food bank staff.” Another participant described an initiative in which the food bank is “training hospital and clinic staff on how to screen for food insecurity, refer to the pantry network, and ideas for programs they can implement onsite, such as a prescriptive pantry or produce distribution.” In addition, partnerships included the development of shared measures as one respondent described, “We are leadership table partners in three accountable communities for health organized around health service areas. Our statewide Accountable Care Organization has adopted food security screening as one of its social determinates of health indicators.”

### 3.2. Food Bank-Healthcare Partnerships Are Leveraging a Variety of Resources and Vested Interests within the Medical Community

Participants discussed various healthcare sector partners collaborating with the food banking sector. Specifically, they mentioned insurance companies, hospitals, and clinical care providers and detailed each partner’s unique motivations and capabilities for engaging with the food banking sector.

#### 3.2.1. Health Insurance Companies

Supporting evidence from interviews

Multiple participants mentioned the health insurance industry as a key partner for the food banking sector. For example, a participant stated:

Other institutions that are starting to be included in this realm of root causes are health insurance companies recognizing they are paying a huge amount for these health disparities. [They are] partnering with hospitals, with food banks, with health insurance companies, saying, “How do we jointly look at this issue, we’re all involved in?”

Participants explained that financial considerations are a powerful motivation for engaging the healthcare industry in food banking partnerships, “because they are the ones that are going to have to pay for a lot of these health inequities as people are going to need healthcare.” Another participant added that the food banking sector may need to make the cost savings apparent to persuade the health insurance industry to partner, stating:

Insurers, as partners, are open to looking at preventative ways through food security programs that then they can see a reduction in cost, again, getting to their motivation. The more that we can help them or put pressure on them to think about improving the health of the community they live, serve, or work in, they think of food insecurity as an opportunity to fund.

#### 3.2.2. Hospitals

Supporting evidence from interviews

Hospitals were another key partner mentioned by food banking stakeholders. Participants mentioned specific examples of initiatives that currently engage hospitals with food banking partners. For instance, one participant mentioned a model in Ohio in which there is “an innovative food pantry within a hospital.” Another participant described other models, noting:

Some hospitals get it, and they change their meal plan and meal services around if their patients have dietary restrictions, so they’re serving and purchasing appropriate food. Similarly, in their cafeterias, where their staff …those who are lowest paid, minimum wage staff may also be food insecure…they’re shopping in their hospital cafeteria.

Participants mentioned that hospitals’ engagement with food banking sector partners had been strengthened by the Affordable Care Act (ACA). One participant stated:

The ACA did help because it was so prevention-focused to give the physicians more time to discuss with their patients things beyond what’s affecting whatever health issue they’re being presented with. I remember…hearing stories about hospitals or physician groups that are more often integrating questions around food security into their overall health assessment questionnaire.

Another participant explained that hospitals’ partnerships with food banking partners had been facilitated by the tax-related flexibility allowed in the ACA, noting:

Nonprofit hospitals became eligible through the ACA to…have food insecurity as one of the…areas where they can get their tax write-off…the ACA opened up a whole new world for hospitals to partner with the charitable food system and food banks …they are now doing those food insecurity screening questions that are referring patients who may not have known that they are eligible for…benefits, including SNAP, but also the food pantries.

Supporting evidence from surveys

Several survey respondents (*n* = 17) mentioned ongoing partnerships between food banks and hospitals. One food bank director shared, “We are building relationships with providers currently,” while another stated, “This is a fast-growing part of our work. We are also partnering with the medical community to distribute healthy food at clinics and hospitals.” Respondents described specific initiatives made possible through collaborations between food banks and hospitals. One respondent mentioned, “One local hospital operates a food pharmacy for people screened as food insecure. We are also partnering with doctor’s offices to provide medically tailored food boxes for patients who are food insecure.” 

#### 3.2.3. Clinical Care Providers

Supporting evidence from interviews

Participants also mentioned clinical care providers, including physicians, as a distinct group of partners within the healthcare sector. Some participants described food banking-healthcare sector partnerships that targeted specific conditions in which they received training from clinical partners. For example, one participant said, “We were just trained to impact 700 people that have diabetes, uncontrollable diabetes, and we’re just trying to improve their Hemoglobin A1C for the next six months.”

Other participants explained the factors that motivate clinical care providers to partner with the food banking sector, specifying that improving health outcomes is a key motivator. One participant summarized clinical care providers’ motivation to engage with the food banking sector as follows:

Food is so inherent in good or bad medical health outcomes. If the doctor prescribes something to a patient where the directions say to take with food, and that patient is food insecure, then I think that the food bank and the medical community would want to come together to make sure that their patients are able to literally follow their doctor’s orders by being able to get food, and particularly healthy food.

## 4. Discussion

This qualitative exploration of food banking stakeholders’ perspectives on food banking and healthcare partnerships reveals evidence of ongoing efforts that target improvements in food security and health outcomes and adds to the growing literature on nutrition-focused food banking [24]. Participants shared the understanding that SDOH are the root causes of both food insecurity and associated health issues. This shared understanding likely underlies food banking stakeholders’ views on the necessity of engaging healthcare sector partners, since food banks alone may not have the resources to address SDOH.

Relatedly, participants stressed the utility of the collective impact framework in facilitating partnerships between the food banking and healthcare systems, especially in setting common goals and deciding on shared measures. Collective impact convenes stakeholders from diverse sectors as partners with a common agenda, synergistic activities, commitment to shared outcomes, continuous communication, and support from a backbone organization [17]. Previous research has highlighted the value of the collective impact approach in addressing complex challenges—typically rooted in poverty and other SDOH—that elude simple, formulaic solutions [25]. This aligns with participants’ observations that collective impact is a useful framework for addressing SDOH in the context of food banking and healthcare partnerships. Importantly, participants emphasized that partners outside of the food banking and healthcare sectors (e.g., housing) are important players in mitigating the impact of food insecurity and related SDOH. Participants noted the need for a backbone organization that convenes food banking and healthcare partners and proposed the types of organizations suitable for such a role. Some interviewees mentioned food banks as being well-positioned to serve as backbone entities. Others identified food policy councils, which convene community members and other local partners to work on food system issues, as “vessels” for connecting healthcare and food bank leaders [26].

Backbone organizations have six primary functions: guiding vision and strategy; supporting aligned activities; establishing shared measurement practices; building public will; advancing policy; and mobilizing funding [27]. Food banks, food policy councils, and healthcare organizations (e.g., hospitals) all have unique strengths to contribute as backbone organizations. For example, food banks may already be set up to develop and track performance metrics for their logistical operations. They may have measurement systems and processes that can be adapted for tracking collective impact outcomes. Food policy councils, on the other hand, may be particularly effective at supporting aligned activities, building public will, and advancing policy due to their inherent focus on multi-sector coalition building and advocacy. Calancie et al. [26] found that activities such as building connections between organizations and advocating for favorable local food policies are commonly undertaken by food policy councils. Lastly, hospitals may also be well-suited to support food banks, as they have access to data on health outcomes that could inform shared measurement goals. Future research can evaluate the types of organizations that are best suited to serve as backbones to food banking and healthcare partnerships.

Participants recognized that the healthcare sector is far from monolithic, both in the resources that different partners can bring to the table, as well as their motivations to do so. This reality has implications for how food banks may frame and message partnership opportunities to healthcare groups. For instance, when working with health insurance companies, food banks can frame their partnerships in light of the long-term, financial returns of investments to improve SDOH. Future research examining the most effective types of messaging to engage varying healthcare partners is warranted.

### Strengths and Limitations

This study contributes to the literature by highlighting the significant partnerships that are taking place at the interface of the food banking and healthcare sectors. It also brings to the forefront the views of food banking stakeholders, a group with unique and relevant, but often understudied, perspectives on food insecurity. The sample size may be considered a limitation of the study. However, interviewees were recruited until we reached saturation and represented the diversity of food banking operation roles, professional perspectives, and geographic locations. Further, the findings from a large national survey were integrated with the interview data, reinforcing the key themes.

## 5. Conclusions

We found evidence of ongoing collaborations between the healthcare and food banking sectors that focus on addressing the root causes of food insecurity. To ensure that partnerships are mutually beneficial, food banking stakeholders should tailor opportunities based on what they know about the interests of the specific type of healthcare partner they are engaging (i.e., providing, administering, or paying for care). Furthermore, to maximize their impact on SDOH, partnerships between the food banking and healthcare systems can be expanded to include additional sectors.

## Figures and Tables

**Table 1:** Themes from Key Stakeholder Interviews (*n* = 10) & Food Bank Director Survey Responses (*n* = 58).

Themes	Sub-Themes	Quote from Interviews (*n* = 10)	Quote from Survey Responses (*n* = 58)
(1) Stakeholders from the healthcare and food banking systems are currently coordinating to achieve a collective impact on addressing SDOH.	1a. Collaborating to Address SDOH	…As people start to look at how we even tackle these root causes, …having connections to people who do SNAP, or health care screenings, or signing up for health insurance within the food pantries…, those types of initiatives are trying to get at some of these root causes. As opposed to just focusing on pounds of food.	We are working with two healthcare groups that are implementing food insecurity screenings. We send SNAP screeners to some of those clinics are working on establishing a referral system to pantries, and a referral program whereby providers can send patient referrals to our Benefits Outreach department through their EMR.
	1b. Applying a Collective Impact Framework	We’ve taken five or six food bank communities and have helped lead them [in] doing a community needs assessment with their partner, helping them to identify what, collectively, they want to make improvements on, what are their measurements, and what role the food bank is going to play now that they have identified those indicators.	We are leadership table partners in three accountable communities for health organized around health service areas. Our statewide Accountable Care Organization has adopted food security screening as one of its social determinants of health indicators.
(2) Food bank-healthcare partnerships are leveraging a variety of resources and vested interests within the medical community.	2a. Health Insurance Companies	Other institutions that are starting to be included in this realm of root causes are …health insurance companies who are recognizing they are paying a huge amount for these health disparities. And partnering with hospitals, with food banks, with health insurance companies, saying, “How do we jointly look at this issue we’re all involved in?”	
	2b. Hospitals	Nonprofit hospitals became eligible through the ACA to…have food insecurity as one of the…areas where they can get their tax write-off…the ACA opened up a whole new world for hospitals to partner with the charitable food system and food banks …they are now doing those food insecurity screening questions that are referring patients who may not have known that they are eligible for…benefits including SNAP but also the food pantries.	One local hospital operates a food pharmacy for people screened as food insecure. We are also partnering with doctor’s offices to provide medically tailored food boxes for food-insecure patients.
	2c. Clinical care providers	We were just trained to impact 700 people that have diabetes, uncontrollable diabetes and we’re just trying to improve their Hemoglobin A1C for the next six months.	

## Data Availability

The data presented in this study are available on request from the corresponding author.
